# Reasons for unplanned hospitalisation in specialist community palliative care: a scoping review

**DOI:** 10.1186/s12904-025-01938-5

**Published:** 2025-12-29

**Authors:** Norah Elvidge, Hannah E. Carter, Melanie Rolfe, Karen Smith, Jane L. Phillips

**Affiliations:** 1 School of Nursing, Queensland University of Technology, Brisbane, QLD 4059 Australia; 2 Silverchain, Melbourne, VIC 3000 Australia; 3 Australian Centre for Health Services Innovation and Centre for Healthcare Transformation, Brisbane, QLD 4059 Australia; 4 School of Public Health and Social Work, Queensland University of Technology, Brisbane, QLD 4059 Australia; 5 School of Population Health, Curtin University, Perth, WA 6102 Australia; 6 Department of Epidemiology and Preventive Medicine, Monash University, Melbourne, VIC 3800 Australia; 7 IMPACCT (Improving Palliative, Aged and Chronic Care through Clinical Research and Translation), University of Technology Sydney, Sydney, NSW 2007 Australia

**Keywords:** Palliative care, Community health services, Hospitalization, Health services accessibility, Specialist community palliative care, Scoping review

## Abstract

**Context:**

Many individuals receiving specialist community palliative care experience unplanned hospitalisations or emergency department presentations. Understanding the reasons for this is essential to developing person-centred solutions.

**Objectives:**

To identify the reasons for unplanned hospitalisations among people receiving specialist community palliative care.

**Methods:**

A PROSPERO (CRD42024495016) registered scoping review reported according to the PRISMA extension for scoping reviews. A search of CINAHL, PubMed, Embase, and Scopus was undertaken for studies published from 2014 to 2025. Two reviewers completed title, abstract and full text screening and data extraction. Extracted reasons for hospitalisation were inductively coded using a content analysis approach, allowing for a descriptive summary of findings.

**Results:**

Thirteen of the 2482 studies identified met the inclusion criteria. Six categories of hospitalisation reason were generated: (1) unrelieved symptoms (16 subcategories), (2) acute event (five subcategories) (3) signs of deterioration, (4) patient or carer distress/fatigue, (5) misunderstanding of the goals of care and (6) other/unknown. Of the sub-categories, dyspnoea (*n* = 11 studies, 85%), pain (*n* = 9, 69%), gastrointestinal symptoms (*n* = 9, 69%) and infection (*n* = 7, 54%) were the most frequently reported reasons for hospitalisation. Comparative studies (*n* = 5) of specialist versus usual or no palliative care showed mixed hospitalisation outcomes, with limited statistical detail.

**Conclusion:**

This scoping review provides insight into patterns of hospitalisation, despite variability in data collection methods. This approach provides a foundation for developing targeted service improvements aimed at supporting individuals to stay at home for more extended periods in the last year of life.

**Supplementary Information:**

The online version contains supplementary material available at 10.1186/s12904-025-01938-5.

## Background

In high-income countries, specialist community palliative care services are increasingly available to support people with palliative care needs [1–4] to manage their complex or refractory symptoms in a home environment [5, 6]. Palliative care can be delivered at home by primary health clinicians, including general practitioners, home nursing services, and nursing home staff. Specialist community palliative care, however, involves the comprehensive assessment and anticipation of physical and psychological symptoms as well as social and spiritual needs, to ensure timely access to care aligned with the patients and their families’ preferences [7]. The need for palliative care is expected to grow globally due to increasing aging populations [8]. Projections suggest a 25% increase in people requiring palliative care by 2048 in the United Kingdom (UK)[9] and 50% increase in Australia by 2035, with a further doubling by 2050 [10]. This growth demands that health services focus on access, outcomes, and experiences of patients and carers in order to design responsive and adaptive services to meet this increase in need [9, 10]. Access is generally defined as the ability of individuals to obtain appropriate healthcare services that align with their needs [11]. Research into unplanned hospitalisations among specialist community palliative care patients is one way of evaluating access to both specialist palliative care and hospital services.

Recent studies have examined hospitalisation rates among patients receiving specialist community palliative care, assessing the impact on care outcomes and healthcare costs [12–14]. However, there is limited evidence on the underlying drivers of unplanned hospital admissions, which is equally essential to informing the design of person-centred, community-based care models. Longhini et al.‘s (2025) scoping review examined reasons for emergency department (ED) presentations in this population; however, the heterogeneity of the included studies limited the findings to predominantly comparing service characteristics [15]. While this evidence is valuable, understanding why patients receiving specialist community palliative care continue to access hospital services requires further examination. Therefore, this scoping review aimed to:

1. Identify and categorise the reasons for unplanned hospital admissions and emergency department presentations (‘hospitalisations’) among people receiving specialist community palliative care; and
2. Compare reasons for unplanned hospitalisation between specialist and non-specialist palliative care cohorts to assess the distinct impact of specialist community palliative care on hospitalisation.

## Methods

This scoping review of quantitative studies was reported in accordance with the PRISMA extension for scoping reviews (supplementary file 1)[16]. Originally registered as a mixed methods systematic review (PROSPERO CRD42024495016), the heterogeneity of the identified quantitative data meant this data was better suited as a scoping review with the intent to identify and map available evidence [17]. A systematic review of the qualitative evidence will be published separately (Elvidge et al., 2025, *under review).*

### Search strategy

A list of base search terms related to home care, palliative care, emergency presentations and hospital admissions was developed and adapted for the included databases, including Embase, CINAHL, PubMed (using the CareSearch palliative care filter) [18] and Scopus. The original searches were conducted on February 8, 2024, and updated on May 20, 2025. Forward and backward citation searching was performed during both search periods. Full details of the search can be found in supplementary file 2.

### Inclusion criteria

This review included English studies published since 2014 to ensure the use of recent evidence, reflecting the significant growth of specialist palliative care in the home over the past decade. Studies were published in a peer-reviewed journal, originating from a high-income economy (as categorised by The World Bank) [19] to ensure comparability of healthcare systems. Included studies employed quantitative data collection of people of any age with a life-limiting condition who were receiving specialist community palliative care services in their home (including nursing homes), where at least 10% of study participants had a recorded reason for hospitalisation.

### Data extraction and analysis

Studies resulting from the search were imported into the bibliographic management software *Covidence*™. The authors (NE, MR, JLP, KS) completed title and abstract screening to determine eligibility. Two reviewers (NE and MR) completed the full-text review individually, and disagreements were resolved via discussion. A third reviewer (JLP) was consulted for remaining conflicts. Two reviewers (NE and MR) completed extraction separately and resolved conflicts via discussion. A proforma template was developed to extract study characteristics and data relevant to the research aims.

The reasons for hospitalisation as reported in each study were independently reviewed by two experienced specialist palliative care nurses (NE and JLP). An inductive approach was employed, beginning with open coding of the spreadsheet data. Through iterative comparison and collaborative discussion, initial codes were refined into categories and subcategories, consistent with the principles of qualitative content analysis [20]. To enable meaningful comparison across studies, we standardised the data by expressing all reported reasons for hospitalisation as proportions. While some studies already presented their data this way, others required conversion from number of hospitalisations. These proportions were subsequently entered into proforma template and organised according to newly developed categories and subcategories. This approach allowed us to identify consistently represented reasons, to rank the top three within each individual study, and enabled a descriptive summary of the findings across studies. The ‘other/unknown’ category was excluded from the top three internal rankings, as its contents are undefined and therefore do not contribute meaningfully to recommendations for targeted care or interventions.

### Quality appraisal

The quality of the included studies was assessed using the JBI critical appraisal tools [21, 22]. Two reviewers independently evaluated each study, discussing its strengths and potential biases based on the checklist criteria. Any disagreements between reviewers were resolved through discussion. These tools do not provide a score for inclusion or exclusion; instead, they guide reviewers in making their determinations [22, 23]. 

## Results

**Fig. 1:**
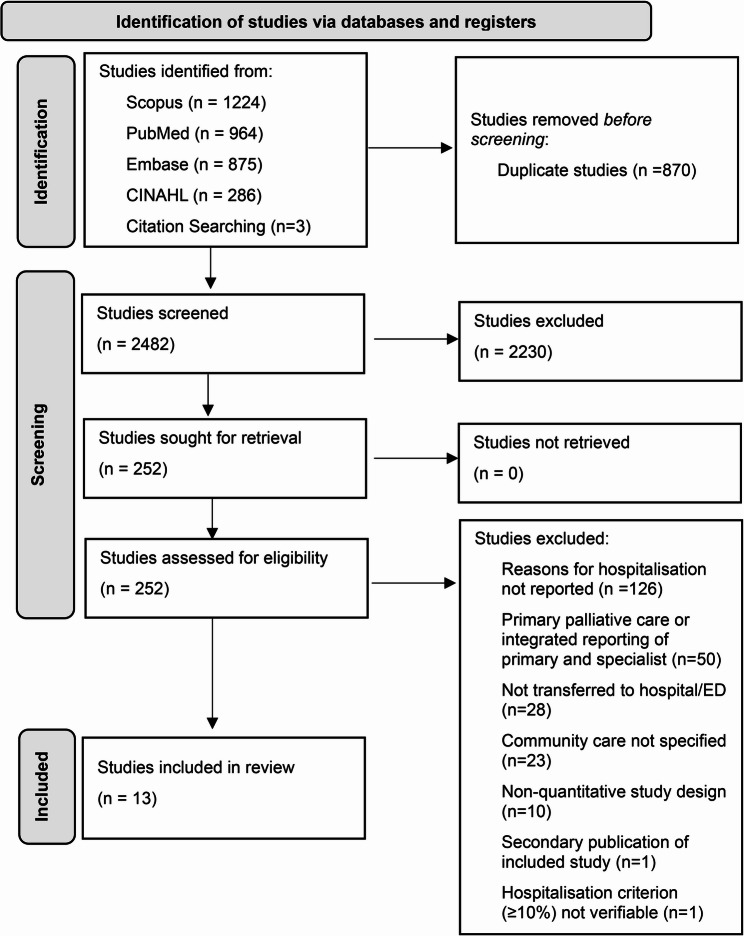
PRISMA flowchart

Of the 3,352 imported citations, 870 duplicates were removed. After screening titles and abstracts, 252 articles underwent full-text review, and 13 studies ultimately met the inclusion criteria (Refer Figure 1). All included studies were deemed to be of acceptable quality for inclusion (See supplementary file 3).

### Study characteristics

The 13 included studies detailed in Table 1were predominantly observational studies such as cross-sectional[24–30], cohort[31–33], and case-control[34], along with two randomised controlled trials (RCTs) [35, 36]. Studies originated from nine high-income countries, with the most represented countries being the United States (US) (*n*= 3)[25, 30, 32], Australia(*n*= 2)[27, 33], and Taiwan (*n*= 2)[31, 34]. The remaining six studies originated from six individual European countries [24, 26, 28, 29, 35, 36]. All of the studies included adults; no paediatric studies were identified. Cancer was the sole diagnosis in six studies[26, 28, 29, 31, 34, 36], One study focused exclusively on people with chronic obstructive pulmonary disease (COPD) [35]. The remaining studies included participants with mixed advanced progressive illnesses [24, 25, 27, 30, 32, 33]. 

**Table 1:** Included study characteristics

Author/Date/ Country and Study Design	Relevant Sample Size and % of Cohort reporting hospitalisation	Demographics	Primary Diagnosis	Service Model	Hours of operation	Types of Service Offerings	Data Source	Comp-arator Data? (Y/*N*)
Martins and Pinto (2023) [24], Portugal, Cross Sectional study	*n* = 187 (intervention – control not reported) 11% of intervention group had ED presentation.	Adult patients Age range < 50 to > 80 55% female	Cancer (82%, *n* = 154), Nervous system disease (13%, *n* = 24) Disease affecting organs (5%, *n* = 9)	Outpatient specialist palliative care (home-linked)	24/7 service options.	Telephone line.	Not specified, presumed chart review of hospital or service records.	Y
DeAngelis and Lowry (2021) [25], United States, Cross Sectional study	*N* = 75 100% admitted to hospital.	Adult patients x̄ age 69.2, 61% female	Cancer (46%, *n* = 35) Dementia (15%, *n* = 11) Heart failure (11%, *n* = 8) Lung disease (11%, *n* = 8) Other (17%, *n* = 13).	Specialist home hospice care	Not reported.	Not reported.	Hospital admission diagnosis	N
Gamblin, Prod’homme (2021) [26], France, Cross Sectional study	*n* = 82 admitted patients, *N* = 142 total study population) 58% admitted to hospital.	Adult patients Age range 26–89, Median age 62 62% female.	Cancer (100%)	Specialist palliative home hospitalisation (France HAD model).	24/7 service options.	Scheduled home visits.	Palliative care service chart review	N
Hsu, Wu (2021) [31], Taiwan^*a*^, Cohort study	*n* = 374 (control) and *n* = 388 (intervention) 440 ED visits in cohort of 762.^*b*^	Adult patients Control vs. Intervention (x̄ age 69.3 vs. 72.6 years) Female: (52.5% vs. 46.9%)	Lung cancer (*n* = 48) Other cancer (*n* = 41)	Specialist community palliative care service.	Comparison of 5-day vs. 7-day service with 24/7 service option.	Scheduled home visits; Telephone line; Education to caregivers.	ED presenting complaint	Y
Cao, Johnson (2020) [32], United States, Cohort study	*n* = 74 (readmitted, *N* = 705 total study population) 10.5% admitted to hospital.	Adult patients, x̄ age 65.7 61% female	Cancer (49%, *n* = 36) Respiratory (5%, *n* = 12) Heart disease (34%, *n* = 25) Kidney disease (3%, *n* = 2) Liver disease (8%, *n* = 6) Other (1% *n* = 1).	Specialist home hospice care.	Not reported.	Not reported.	Hospital chart review	N
Scheerens, Pype (2020) [35], Belgium, Phase II Randomised Control Trial	*n* = 19 (control) and *n* = 20 (intervention) 46% with admission at baseline, percentage ranged throughout checkpoints from 10–26%.	Adult patients Control vs. Intervention (Median age 67 vs. 67.5 years) Female: (42% vs. 45%)	Chronic Obstructive Pulmonary Disease (COPD) (100%)	Specialist community palliative care service.	Not reported	Scheduled home visits.	Hospital admission diagnosis	Y
Jessop, Fischer (2018) [27], Australia, Cross Sectional study	*N* = 88 97 hospital admissions in cohort.	Adult patients x̄ age 69 41% female	Cancer (82% of admissions *n* = 85) Non-malignancy (15% of admissions, *n* = 16)	Specialist community palliative care service.	24/7 service options.	Scheduled home visits; Telephone line; Emergency home visits.	Hospital chart review, interdisciplinary team meetings, discussion with specialist nurse.	N
Kao, Liu (2018) [34], Taiwan, Case-Control study	*N* = 65 100% had ED presentation	Adult patients x̄ age 73 46% female	Cancer (100%)	Specialist hospice shared-care model offered in patient home.	Monday to Friday, 8am to midnight.	Scheduled home visits; Telephone line.	Hospital chart review	N
Skov Benthien, Nordly (2018) [36], Denmark, Randomised Control Trial	*n* = 160 (control) and *n* = 162 (intervention) 78% of intervention group had hospital admission (*n* = 127).	Adult patients Control vs. Intervention (x̄ age 65 vs. 66 years) Female (82% vs. 83%)	Cancer (100%)	Specialist community palliative care service and Specialist home hospice care.	Not reported.	Scheduled home visits; Psychologist support for patient and carer.	Hospital admission diagnosis	Y
Kaiser, Rudloff (2017) [28], Germany, Cross Sectional study	*n* = 24 admitted (*N* = 73 total study population) 33% admitted to hospital.	Adult patients 87.6% over 65 years 44% female	Haematological malignancies (100%)	Specialist community palliative care service.	Not reported.	Scheduled home visits; Telephone line.	Hospital admission diagnosis	N
Spilsbury, Rosenwax (2017) [33], Australia, Cohort study	*n* = 8050 (control) and *n* = 3825 (intervention) 24.9% with 1 admission, 17.8 with 2 and 30.2 with ≥ 3 admissions (within intervention group)	Adult patients > 20 years Majority male (Female 0.96 h, 0.93–0.99 95% CI).	Cancer (62%, *n* = 7411) Heart failure (17%, *n* = 2019) Renal failure (10% *n* = 1145) COPD (9% *n* = 1094) Liver failure (2% *n* = 206)	Specialist community palliative care service.	24/7 service options.	Scheduled home visits; Telephone line; Emergency home visits; Respite; Counselling; Linkage to other service; Pall care nurse consultancy in nursing homes.	ED presenting complaint	Y
Mercadante, Masedu (2016) [29], Italy, Cross Sectional study	*n* = 138 (admitted, *N* = 550 total study population) 25% admitted to hospital.	Adult patients x̄ age 69 40.6% female	Cancer (100%)	Specialist community palliative care service.	24/7 service options.	Scheduled home visits; Telephone line; Emergency home visits.	Palliative care service chart review	N
Batchelor (2015) [30], United States, Cross Sectional study	*N* = 111 100% had ED presentation	Adult patients x̄ age 77	Cancer Dementia/Frailty Respiratory disease Heart/vascular disease Adult failure to thrive (n = not reported)	Specialist community palliative care service.	24/7 service options.	Scheduled home visits; Telephone line.	Palliative care service chart review	N

^*a*^ Despite a total study population of *N* = 762, demographic and primary diagnosis data was only available for the *n* = 89 patients hospitalised for dyspnoea

^*b*^ While the percentage of the cohort with a hospitalisation was not reported, the total number of admissions was provided. Based on this number, it is assumed that admissions involved > 10% of the cohort

Service models were predominantly described as specialist community palliative care services (*n*= 8)[27–31, 33, 35, 36]. One study referred to an outpatient specialist palliative care service for patients living at home [24]. Five studies described home hospice (*n* = 4)[25, 32, 34, 36] or hospital-at-home services (*n* = 1)[26] that align with specialist community palliative care, although the terminology reflects country-specific conventions. Seven studies reported 24/7 service options[24, 26, 27, 29–31, 33], one reported limited service hours[34], and the remaining studies (*n* = 5) did not provide substantial information on hours of operation. Where service descriptions were available, most (*n* = 11) included scheduled home visits (*n*= 10)[26–31, 33–36], and/or telephone support (*n*= 8)[24, 27–31, 33, 34]. Three reported offering emergency home visits [27, 29, 33]. Additional services by a small number of studies included psychological support[36], respite care[33], carer education[31], and referrals to other services [33]. Only Spilsbury et al. (2018)included nursing home residents in its stated study population[33].

The reasons for hospitalisation were predominantly extracted from hospital or palliative care service chart reviews (*n*= 7)[24, 26, 27, 29, 30, 32, 34], and a smaller number from admission diagnosis (*n*= 4)[25, 28, 35, 36], or the emergency department presentation (*n*= 2)[31, 33]. Five studies included relevant comparator data [24, 31, 33, 35, 36] where the control was ‘usual’, or non-specialist palliative care, and the interventions involved establishing or enhancing existing community palliative care services.

### Reasons for hospitalisation

**Fig. 2:**
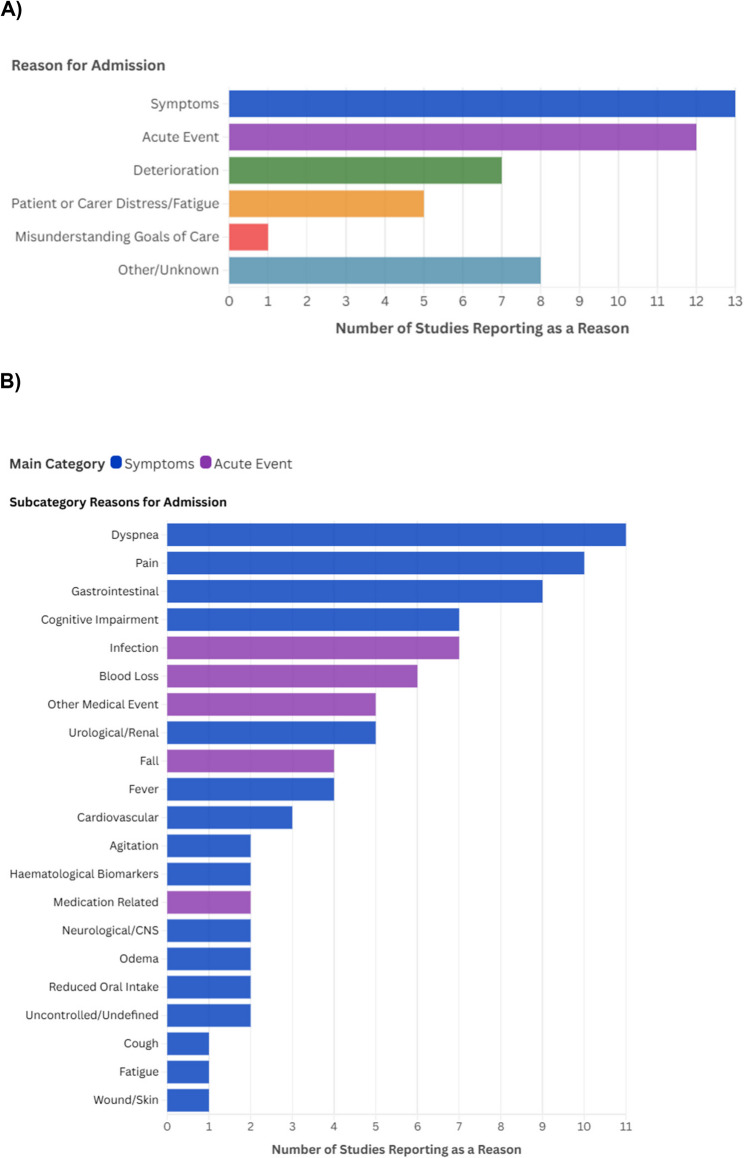
Categories of reported reasons. (**A**) Ranked distribution of categories across 13 studies. (**B**) Ranked distribution of subcategories across 13 studies

The reasons for hospitalisation fell into six main categories: (1) unrelieved symptoms (‘symptoms’) (16 subcategories), (2) acute event (five subcategories) (3) signs of deterioration (‘deterioration’), (4) patient or carer distress/fatigue, (5) misunderstanding of the goals of care and (6) other/unknown. Table 2 outlines the distribution of categorised reasons in each study, and a ranked summary of the categories and subcategories is depicted in Fig. 2. More detailed descriptions of the reasons for hospitalisation, as well as reported admission proportions, are available in supplementary file 4.

**Table 2:** Reasons for hospitalisation among specialist community palliative care populations

Categories	**Subcategories**	Martins and Pinto (2023) [24]	Hsu, Wu (2021) [31]	Scheerens, Pype (2020) [35]	Skov Benthien, Nordly (2018) [36]	Spilsbury, Rosenwax (2017) [58]	DeAngelis and Lowry (2021) [25]	Gamblin, Prod'homme (2021) [26]	Cao, Johnson (2020) [32]	Jessop, Fischer (2018) [27]	Kao, Liu (2018) [34]	Kaiser, Rudloff (2017) [28]	Mercadante, Masedu (2016) [29]	Batchelor (2015) [30]
Symptoms	*Dyspnoea*	✓*	✓*	✓*	✓*	✓*		✓*		✓*	✓*	✓﻿	✓*	✓﻿
	*Pain*	✓	✓*		✓*	✓*		✓﻿		✓*	✓﻿*	✓*	✓﻿	✓﻿
	*Gastrointestinal*	✓	✓*		✓﻿	✓﻿		✓﻿		✓﻿	✓﻿	✓﻿	✓﻿	
	*Cognitive Impairment*		✓﻿		✓﻿			✓﻿		✓﻿		✓*	✓﻿	✓﻿
	*Urological/Renal*	✓			✓﻿							✓﻿	✓﻿	✓*
	*Fever*	✓*	✓﻿		✓﻿								✓﻿	
	*Cardiovascular*					✓*		✓﻿				✓﻿		
	*Uncontrolled/Undefined*						✓*		✓*		✓﻿			
	*Neurological/CNS*				✓﻿								✓*	
	*Oedema*	✓										✓﻿		
	*Agitation*	✓﻿										✓﻿		
	*Haematological Biomarkers*		✓﻿		✓﻿									
	*Reduced Oral Intake*	✓﻿			✓﻿									
	*Cough*	✓﻿												
	*Fatigue*	✓*												
	*Wound and Skin Conditions*											✓﻿		
Acute Events	*Infection*			✓*	✓﻿			✓*		✓﻿	✓*	✓﻿		✓﻿
	*Blood Loss*	✓﻿	✓﻿					✓﻿			✓﻿	✓﻿	✓﻿	
	*Other Medical Event*		✓﻿				✓*	✓﻿	✓*					✓*
	*Fall*		✓﻿		✓﻿					✓﻿				✓*
	*Medication Related*						✓*					✓﻿		
Deterioration	n/a	✓﻿		✓*	✓*			✓*		✓﻿	✓﻿	✓*		
Patient/Carer Distress/Fatigue	n/a				✓﻿			✓﻿	✓﻿	✓*			✓*	
Misunderstanding Goals of Care	n/a								✓*					
Other/Unknown	n/a	✓﻿		✓﻿	✓﻿			✓﻿		✓﻿		✓﻿	✓﻿	✓﻿

*NB*: A tick (✓﻿) indicates the reason was reported in the study; ✓﻿* denotes this was one of the top three reasons within that study

#### Distribution of hospitalisation reasons

Of the six categories, symptoms were the most frequently reported and were a reason for unplanned hospitalisation in all studies, followed by acute events (*n*= 12, 92%)[24–32, 34–36], deterioration (*n*= 7, 54%)[24, 26–28, 34, 36], and patients’ or carers’ experiences of distress and/or fatigue (*n*= 5, 38%)[26, 27, 29, 32, 36]. Within this category, four of the five studies explicitly referred to *carer* distress or fatigue [26, 27, 29, 32]. Misunderstanding of the goals of care was noted in one study (8%)[32]. However, while not identified as a primary reason for hospitalisation, in DeAngelis & Lowry (2021), it was reported that patients or carers had a poor understanding of palliative care in 16% of unplanned admissions [25]. 

#### Subcategory results and internal ranking

Within the symptom subcategories, dyspnoea was the most frequently reported reason for hospitalisation, reported in 11 (85%) studies[24, 26–31, 33–36],followed by unrelieved pain [24, 26–29, 31, 33, 34, 36] and gastrointestinal symptoms[24, 26–29, 31, 33, 34, 36], which were each reported in nine (69%) studies. Infection (*n* = 7, 54%) [26–28, 30, 34–36],blood loss stemming from tumour complications, gastrointestinal bleeds or undefined haemorrhage (*n*= 6, 46%)[24, 26, 28, 29, 31, 34], and other acute medical events (*n* = 5, 38%)[25, 26, 30–32] were the most reported acute event subcategories. The top three reasons for hospitalisation within each study, identified through internal ranking by proportion (refer to Table 2), were dyspnoea (*n*= 8, 62%)[24, 26, 27, 29, 31, 33, 34, 36], pain (*n*= 6, 46%)[27, 28, 31, 33, 34, 36], and deterioration (*n*= 4, 31%)[26, 28, 35, 36].

### Comparison of specialist and non-specialist/no palliative care

Comparative data were reported in five studies; two RCTs [35, 36] and three observational studies [24, 31, 33]. Where statistically significant results were found, the RCTs reported that specialist palliative care interventions were associated with increased hospitalisation rates, either for specific reasons or in general [35, 36]. Skov Benthien et al. (2018) identified two specific significant reasons; worsened general health (*p* = 0.04) and unmanageable care at home (*p*= 0.01)[36], which were categorised as deterioration and patient or carer distress or fatigue. Other reasons for hospitalisation in this study were not statistically significant between cohorts [36]. Scheerens et al. (2020) reported a statistically significant difference in overall hospitalisations between groups (*p* = 0.03), however, statistical comparisons for individual reasons for admission were not reported [35]. 

Observational studies generally found that specialist community palliative care was associated with a reduction in overall hospital admissions. However, its impact on specific reasons for hospitalisation varied. Hsu et al. (2021) reported that a specialist community palliative care program for cancer patients targeted to relieve dyspnoea led to a 30.7% (*p* < 0.05) reduction in dyspnoea-related hospitalisations compared with usual palliative care services [31]. Spilsbury et al. (2017) reported that individuals receiving specialist community palliative care had a 50% lower ED presentation rate overall (95% CI: 47%–53%), and were less likely to present to hospital with dyspnoea, but more likely to report pain and nausea (no statistical data provided) [33]. While Martins and Pinto (2023) found that implementing a specialist 24/7 phone service reduced hospital admissions overall and shifted the top reasons from pain, dyspnoea, and nausea/vomiting to dyspnoea, fever, and asthenia post-implementation, no inferential statistics were reported [24]. 

## Discussion

This scoping review found the most common reasons for hospitalisation among specialist community palliative care patients were related to unrelieved symptoms, acute medical events and deteriorating health, as well as a smaller number of studies reporting psychosocial reasons such as patient or carer distress and fatigue or misunderstanding of palliative care objectives. Methods for classifying hospitalisation reasons varied across studies, often relying on diagnostic codes, with limited evidence available to compare outcomes in cohorts receiving either non-specialist, or no palliative care.

### Symptom-related findings

Unrelieved symptoms, particularly dyspnoea, pain, gastrointestinal symptoms and cognitive impairment, were the most frequent reasons for unplanned symptom-related hospitalisation. This aligns with existing evidence of the burden of symptoms in specialist palliative care [37–40]. Dyspnoea is prevalent in both chronic respiratory (e.g., COPD, interstitial lung disease) and non-respiratory diseases (heart failure and pulmonary secondaries, cancer-related cachexia) and tends to increase as the end-of-life approaches [38, 41, 42]. For over a decade, data from palliative care services have consistently demonstrated a high prevalence of moderate to severe dyspnoea with limited effective treatment[41, 43], highlighting the persistent challenges in managing refractory dyspnoea among patients with palliative care nearing the end-of-life.

Some of the symptom subcategories that were highly prevalent overall, such as cognitive impairment and gastrointestinal symptoms, only appeared once as a top three reason in the internal rankings [28, 31]. The descriptive data in this scoping review offer limited explanation for this. However, existing research indicates that gastrointestinal symptoms such as nausea, vomiting, constipation, diarrhoea, and ascites frequently co-occur with other symptoms or emerge during periods of patient instability [44, 45]. This may result in underreporting in hospital settings, where more dominant clinical issues are prioritised [44]. 

### Psychosocial factors

In this scoping review, the ‘patient or carer distress/fatigue’ category included both patient and carer-related factors, but most codes related to carer distress. Carer wellbeing has previously been linked to well-coordinated and continuous specialist community palliative care [46]. And carer distress has been linked to several factors reflected in the findings from this scoping review, including symptoms, acute events, and deterioration, particularly when they feel unprepared [38, 47]. The definition of an ‘emergency’ may evolve when approaching the end of life, meaning symptoms and events are often managed conservatively outside of acute care [38]. However, this depends on carers having the physical, emotional, spiritual and financial capacity to manage care confidently at home, aided and supported by appropriate care planning and practical assistance [38, 48, 49]. 

Recent evidence suggests symptom management can be improved by addressing unmet social or spiritual needs [37, 50]. However, the results of this scoping review found psychosocial factors were considerably less reported, suggesting these unmet needs may not be adequately captured in the quantitative data. For instance, a symptom may be reported as the reason for hospitalisation, but the underlying driver could have been carer fatigue or limited service ability to address that symptom in the community environment. This is contradictory to qualitative observational studies that often highlight psychosocial factors for patients and carers alike, such as inability to cope at home, service-related issues such as limited hours and poor transitions from hospital, and societal attitudes toward palliative care as key contributors to unplanned hospitalisation [51–53]. Addressing refractory symptoms or acute events in community palliative care requires whole of sector strategies that encompasses physical, social, and spiritual needs. Emerging models of care, such as one developed in 2022 by Chapman et al. presents a holistic, multi-disciplinary approach to symptom management that emphasises the importance of collaboration between services, clinicians, patients, and families to achieve meaningful outcomes [37]. Embedding this approach within specialist palliative care acknowledges the complex interplay of factors influencing health service access and could support more responsive patient-centred care.

### Comparative findings

Comparative findings in this scoping review were consistent with existing evidence. Observational research has frequently reported fewer hospitalisations associated with specialist community palliative care[54–60], whereas the experimental research is considerably mixed, with some studies demonstrating reductions[61–63], and others showing no significant effect [36, 64–67]. The significant findings from two RCT’s included in this scoping review reported increases in hospitalisation when implementing specialist community palliative care [35, 36]. Indeed, a reduction in hospitalisations has yet to be confirmed through meta-analyses, which, as with this scoping review, have been limited by heterogeneity in interventions and data collection [12, 46]. Further research in this area will strengthen the evidence base for specialist community palliative care, enabling statistical comparisons across studies and supporting future meta-analyses.

### Implications for future research and practice

Identifying the most common reasons for hospitalisation offers valuable direction for future research and provides a baseline for evaluating the impact of targeted service improvements. The results may inform distribution of resources towards early recognition and management of these common reasons for hospitalisation and encourage better coordination between acute services and home-based palliative care.

The data collected in this scoping review primarily reflect access to hospital services measured through hospital diagnostic codes. Further consideration of clinical and psychosocial needs and collaboration between healthcare providers, patients, and families is needed to enact appropriate support which may mitigate unplanned hospitalisations [37]. Additionally, The lack of comparative data limits conclusions about how specialist care specifically influences reasons for hospitalisation, and more robust research encompassing experimental comparative study designs, is needed to understand how to prevent avoidable hospitalisations and better tailor care to the complex needs of people with life-limiting conditions at home.

### Strengths and limitations

The strength of this scoping review is that the included studies drew on a diverse range of hospital data sources, including emergency departments, inpatient admissions, and specialist community palliative care records, offering a broad perspective. Despite this significant strength, this review has several limitations. Restricting inclusion to high-income countries improved comparability but introduced geographic gaps and may have limited the number of included studies.Data comparability was limited by methodological heterogeneity and inconsistent reporting across studies. While some studies allowed multiple reasons per hospitalisation, others only permitted one. As a result, the analysis focused on the prevalence and internal ranking of reasons for admission, rather than raw admission rates, and meta-analysis was not possible, which has been similarly acknowledged in other recent related review as a limitation affecting the comparability of data [12, 15]. 

While seven studies described 24/7 services and one other reported service hours, the service availability in the remaining studies was unclear. Providing 24/7 care is increasingly seen as a standard of quality specialist community palliative care, particularly in regard to reducing hospital admissions [50]. Moreover, reporting on the types of services offered was limited, and although studies commonly referenced telephone lines and scheduled home visits, few provided comprehensive service lists, suggesting key data may be missing for proper comparative analysis. Future literature in this field should aim to include more detailed descriptions of the services involved to facilitate more meaningful comparison.

Most data were drawn from routinely collected admission records, obscuring potential social or contextual factors. To explore these factors in more depth, a separate review of qualitative studies was undertaken and has been reported elsewhere (Elvidge et al., 2025, *under review).* Lastly, while categories and subcategories were developed inductively by two palliative care nurses, some subjectivity was involved in their application.

## Conclusion

This scoping review summarises the most prevalent reasons for hospitalisation among patients receiving specialist community palliative care, based on clinical data sources such as admission records and diagnostic codes. It also highlights conflicting evidence on whether these common reasons are increased or reduced by such services, compared to generalist community palliative care. The findings suggest that addressing unplanned hospitalisations requires person-centred research that examines the experiences of patients, carers, and healthcare providers, alongside more robust comparative studies to guide service improvements and tailor care to complex needs at home.

## Supplementary Information

Supplementary Material 1.

Supplementary Material 2.

Supplementary Material 3.

Supplementary Material 4.

## Data Availability

No datasets were generated or analysed during the current study.

## Abbreviations

COPD: Chronic obstructive pulmonary disease
ED: Emergency department
RCT: Randomised controlled trial
UK: United Kingdom
US: United States
