# Supplementary material for: Reasons for unplanned hospitalisation in specialist community palliative care: a scoping review
Source: BMC Palliat Care. 2025 Dec 29;24:304. doi: 10.1186/s12904-025-01938-5 (PMC12751587; doi:10.1186/s12904-025-01938-5)
Supplement: Supplementary file 2 — Supplementary Material 2. [file 12904_2025_1938_MOESM2_ESM.docx]

# Supplemental File 2

**Search terms for the Scoping Review**

| "community care", "care at home", "home health nursing", "home care", "home based care", "home nursing", "home care services", "home health care", "community nursing" |
| --- |
| "palliative care", "end of life care", “terminal care", "supportive care", "palliative therapy", "hospice care", "life limiting disease", "life limiting illness", "life limiting condition", "terminal illness", "terminal disease", "advanced disease", "advanced malignancy" |
| "Hospital admission", "hospital readmission", "unplanned hospital admission", "unplanned hospital readmission", hospitali?ation, "hospital stay", "acute admission", "emergency department", ED, "accident and emergency", A&E, "inpatient ward", "hospital use", "hospital presentation" |
| 1 and 2 and 3 |
| Limiters - Publication Date: 20140101-20250520 and English language |
| *Note: slight variations in search terms were used to adapt to different databases.* |
