# Supplementary material for: Reasons for unplanned hospitalisation in specialist community palliative care: a scoping review
Source: BMC Palliat Care. 2025 Dec 29;24:304. doi: 10.1186/s12904-025-01938-5 (PMC12751587; doi:10.1186/s12904-025-01938-5)
Supplement: Supplementary file 3 — Supplementary Material 3. [file 12904_2025_1938_MOESM3_ESM.docx]

# Supplemental File 3

**JBI Quality Assessment**

Guidance for the quality assessment questions was derived from the JBI critical appraisal tool guidelines for RCT (1), cohort (2), cross-sectional (3) and case control (4) studies.

| Randomised Controlled Trials | | | | | | | | | | | | | |
| --- | --- | --- | --- | --- | --- | --- | --- | --- | --- | --- | --- | --- | --- |
| **Study** | **Q1** | **Q2** | **Q3** | **Q4** | **Q5** | **Q6** | **Q7** | **Q8** | **Q9** | **Q10** | **Q11** | **Q12** | **Q13** |
| Skov Benthien, Nordly (36) | Yes | No | Yes | No | No | Yes | Yes | Yes | Yes | Yes | Yes | Yes | Yes |
| Scheerens, Pype (35) | Yes | No | Yes | No | No | No | Yes | Yes | Yes | Yes | Yes | Yes | Yes |
| Cohort Study | | | | | | | | | | | | | |
| Cao, Johnson (32) | Yes | N/A | N/A | Yes | Yes | N/A | Yes | Yes | Yes | Yes | Yes |  |  |
| Spilsbury, Rosenwax (33) | Yes | Yes | Yes | Yes | Yes | Yes | Yes | Yes | N/A | N/A | Yes |  |  |
| Hsu, Wu (31) | Yes | Yes | Yes | Yes | Yes | N/A | Yes | Yes | Yes | N/A | No |  |  |
| Cross Sectional Study | | | | | | | | | | | | | |
| Martins and Pinto (24) | Yes | Yes | Yes | Yes | No | No | Yes | No |  |  |  |  |  |
| DeAngelis and Lowry (25) | Yes | Yes | N/A | Yes | Yes | No | No | N/A |  |  |  |  |  |
| Gamblin, Prod'homme (26) | Yes | Yes | Yes | Yes | N/A | N/A | Yes | Yes |  |  |  |  |  |
| Jessop, Fischer (27) | Yes | Yes | Yes | Yes | N/A | N/A | Yes | Yes |  |  |  |  |  |
| Kaiser, Rudloff (28) | Yes | Yes | Yes | Yes | N/A | N/A | No | N/A |  |  |  |  |  |
| Mercadante, Masedu (29) | Yes | Yes | Yes | Yes | No | No | Yes | Yes |  |  |  |  |  |
| Batchelor (30) | Yes | No | Yes | Yes | No | No | Yes | Yes |  |  |  |  |  |
| Case Control | | | | | | | | | | | | | |
| Kao, Liu (34) | Yes | Yes | Yes | Yes | Yes | Yes | N/A | Yes | Yes | Yes |  |  |  |

1. Barker TH, Stone JC, Sears K, Klugar M, Tufanaru C, Leonardi-Bee J, et al. The revised JBI critical appraisal tool for the assessment of risk of bias for randomized controlled trials. JBI Evidence Synthesis. 2023;21(3):494-506. [Https://doi.org/10.11124/JBIES-22-00430](https://doi.org/10.11124/JBIES-22-00430).

2. Barker TH, Hasanoff S, Aromataris E, Stone JC, Leonardi-Bee J, Sears K, et al. The revised JBI critical appraisal tool for the assessment of risk of bias for cohort studies. JBI Evidence Synthesis. 2025;23(3):441-53. [Https://doi.org/10.11124/JBIES-24-00103](https://doi.org/10.11124/JBIES-24-00103).

3. Moola S, Munn Z, Tufanaru C, Aromataris E, Sears K, Sfetcu R, et al. Systematic reviews of etiology and risk. JBI Manual for Evidence Synthesis. 2020. [Https://doi.org/https://doi.org/10.46658/JBIMES-24-06](https://doi.org/https://doi.org/10.46658/JBIMES-24-06).

4. Munn Z, Barker T, Moola S, Tufanaru C, Stern C, McArthur A, Stephenson M, Aromataris E. Methodological quality of case series studies, JBI Evidence Synthesis, doi: 10.11124/JBISRIR-D-19-00099
