# Supplementary material for: Reasons for unplanned hospitalisation in specialist community palliative care: a scoping review
Source: BMC Palliat Care. 2025 Dec 29;24:304. doi: 10.1186/s12904-025-01938-5 (PMC12751587; doi:10.1186/s12904-025-01938-5)
Supplement: Supplementary file 4 — Supplementary Material 4. [file 12904_2025_1938_MOESM4_ESM.docx]

# Supplementary File 4

Complete results of data extraction; categorisation of reasons into categories and subcategories and reported and calculated percentages of hospitalisations attributed to each reason, as well as significant results, where reported.

| **Author, date** | **Reasons for hospitalisation - exact primary study wording** | **Category** | **Subcategory (if applicable)** | **Hospitalisations attributed to reason^a,b^ (%)** | **Significance SPC vs non-SPC cohorts** |
| --- | --- | --- | --- | --- | --- |
| **Martins and Pinto (2023)** | Dyspnoea | Unrelieved Symptoms (‘Symptoms’) | Dyspnoea | 22 | Statistical significance was set at *p* < 0.05; however p-values were not reported, and no correction for multiple comparisons was noted, limiting interpretability of the statistical findings. Before SPC, the top three reasons were Pain (30%), Dyspnoea (21%) and Vomiting (9%). |
|  | Fever | Symptoms | Fever | 19 |  |
|  | Asthenia | Symptoms | Fatigue | 16 |  |
|  | Pain | Symptoms | Pain | 14 |  |
|  | End of life | Signs of Deterioration (‘Deterioration’) |  | 11 |  |
|  | Vomiting | Symptoms | Gastrointestinal | 6 |  |
|  | Oedema | Symptoms | Oedema | 4 |  |
|  | Blood loss | Acute Event | Blood loss | 2 |  |
|  | Agitation | Symptoms | Agitation | 2 |  |
|  | Other | Other/Unknown |  | 2 |  |
|  | Cough | Symptoms | Cough | 1 |  |
|  | Urinary | Symptoms | Urological/Renal | 1 |  |
|  | Food refusal | Symptoms | Reduced oral intake | 1 |  |
| **DeAngelis and Lowry (2021)** | Unanticipated new medical issue | Acute Event | Other medical event | 73 |  |
|  | Uncontrolled symptoms | Symptoms | Uncontrolled/Undefined | 45 |  |
|  | Medication related problem | Acute Event | Medication related | 57 |  |
| **Gamblin, Prod'homme (2021)** | Dyspnoea | Symptom | Dyspnoea | 21 |  |
|  | Infection | Acute Event | Infection | 13 |  |
|  | Intervention requiring technical expertise | Deterioration |  | 11 |  |
|  | Pain | Symptom | Pain | 10 |  |
|  | Digestive event | Symptom | Gastrointestinal | 10 |  |
|  | Delirium | Symptom | Cognitive impairment | 9 |  |
|  | Other | Other/Unknown |  | 9 |  |
|  | Caregiver burnout | Patient or carer distress/fatigue |  | 6 |  |
|  | Decline in general condition | Deterioration |  | 6 |  |
|  | Iatrogenic event | Acute Event | Other medical event | 3 |  |
|  | Cardiovascular event | Symptom | Cardiovascular | 1 |  |
|  | Hemorrhage | Acute Event | Blood loss | 1 |  |
| **Hsu, Wu (2021)^c^** | Gastrointestinal symptoms | Symptoms | Gastrointestinal | 20 |  |
|  | Dyspnoea | Symptoms | Dyspnoea | 19 | Less prevalent in the intervention group (*p*<0.05) |
|  | Pain | Symptoms | Pain | 18 |  |
|  | Fever | Symptoms | Fever | 15 |  |
|  | Altered mental status | Symptoms | Cognitive impairment | 12 |  |
|  | Catheter-related events | Acute Event | Other medical event | 8 |  |
|  | Anemia | Symptoms | Haematological biomarker | 4 |  |
|  | Fell down | Acute Event | Fall | 2 |  |
|  | Tumor bleeding/complication | Acute Event | Blood loss | 1 |  |
| **Cao, Johnson (2020)** | Unanticipated new medical issue | Acute event | Other medical event | 45 |  |
|  | Uncontrolled symptoms | Symptoms | Uncontrolled/Undefined | 34 |  |
|  | Misunderstanding of hospice status | Misunderstanding goals of care |  | 16 |  |
|  | Caregiver distress | Patient or carer distress/fatigue |  | 5 |  |
| **Scheerens, Pype (2020)** | Acute exacerbation of COPD/COPD related symptoms^d^ | Symptoms | Dyspnoea | 72 | Overall, there were fewer hospitalisations in the *control* group compared to the intervention (*p*=0.03). |
|  | Coma | Deterioration |  | 11 |  |
|  | Other | Other |  | 11 |  |
|  | Pneumonia | Acute Event | Infection | 6 |  |
| **Jessop, Fischer (2018)** | Pain | Symptoms | Pain | 27 |  |
|  | Patient/family distress or request for admission | Patient or carer distress/fatigue |  | 25 |  |
|  | Breathlessness | Symptoms | Dyspnoea | 16 |  |
|  | Confusion | Symptoms | Cognitive impairment | 7 |  |
|  | Accident/fall | Acute Event | Fall | 6 |  |
|  | Infection | Acute Event | Infection | 6 |  |
|  | Other | Other/Unknown |  | 5 |  |
|  | Nausea or vomiting | Symptoms | Gastrointestinal | 5 |  |
|  | Constipation or diarrhea | Symptoms | Gastrointestinal | 2 |  |
|  | Comorbidities | Deterioration |  | 1 |  |
| **Kao, Liu (2018)** | Pain | Symptoms | Pain | 72 |  |
|  | Dyspnea | Symptoms | Dyspnoea | 48 |  |
|  | Infection or Fever | Acute Event | Infection | 48 |  |
|  | Change of consciousness | Deterioration |  | 25 |  |
|  | Nausea or Vomiting | Symptoms | Gastrointestinal | 18 |  |
|  | Gastrointestinal bleeding | Acute Event | Blood loss | 18 |  |
|  | Composite distressing condition (two of three symptoms: dyspnea, change of consciousness, and gastrointestinal bleeding) | Symptoms | Uncontrolled/Undefined | 18 |  |
|  | Constipation | Symptoms | Gastrointestinal | 12 |  |
| **Skov Benthien, Nordly (2018)** | Worsened general health | Deterioration |  | 22 | More prevalent in the intervention group (*p*=0.0436) |
|  | Pain | Symptoms | Pain | 20 |  |
|  | Dyspnoea | Symptoms | Dyspnoea | 18 |  |
|  | Dehydration | Symptoms | Reduced oral intake | 10 |  |
|  | Cancer progression | Deterioration |  | 9 |  |
|  | Unmanageable home situation | Patient or carer distress/fatigue |  | 8 | More prevalent in the intervention group (*p*=0.0119) |
|  | CNS Symptoms | Symptoms | Neurological/CNS | 8 |  |
|  | Need for palliative/supportive care | Deterioration |  | 8 |  |
|  | Fever | Symptoms | Fever | 6 |  |
|  | Confusion | Symptoms | Cognitive impairment | 6 |  |
|  | Pneumonia | Acute Event | Infection | 6 |  |
|  | Constipation | Symptoms | Gastrointestinal | 5 |  |
|  | Other Infection | Acute Event | Infection | 5 |  |
|  | Ascites | Symptoms | Gastrointestinal | 5 |  |
|  | Vomiting | Symptoms | Gastrointestinal | 5 |  |
|  | Fall | Acute Event | Fall | 5 |  |
|  | Anemia | Symptoms | Haematological biomarkers | 4 |  |
|  | Neutropenia | Symptoms | Haematological biomarkers | 3 |  |
|  | Psychological distress | Patient or carer distress/fatigue |  | 3 |  |
|  | Acute abdomen | Symptoms | Pain | 3 |  |
|  | Other non-cancer causes | Other/Unknown |  | 3 |  |
|  | Swallowing difficulty | Symptoms | Reduced oral intake | 2 |  |
|  | Diarrhea | Symptoms | Gastrointestinal | 2 |  |
|  | Uraemia or hydronephrosis | Symptoms | Urological/Renal | 2 |  |
|  | Urinary tract infection | Acute Event | Infection | 2 |  |
| **Kaiser, Rudloff (2017)** | Impaired general status, weakness | Deterioration |  | 54 |  |
|  | Pain | Symptoms | Pain | 29 |  |
|  | Psychological symptoms (consciousness, confusion) | Symptoms | Cognitive impairment | 21 |  |
|  | Infections | Acute Event | Infection | 17 |  |
|  | Dyspnoea | Symptoms | Dyspnoea | 13 |  |
|  | Restlessness | Symptoms | Agitation | 13 |  |
|  | Nausea, vomiting | Symptoms | Gastrointestinal | 13 |  |
|  | Incontinence/UTI | Symptoms | Urological/Renal | 13 |  |
|  | Bleeding | Acute Event | Blood loss | 8 |  |
|  | Drug application problems | Acute Event | Medication related | 8 |  |
|  | Unknown | Other/Unknown |  | 8 |  |
|  | Constipation | Symptoms | Gastrointestinal | 4 |  |
|  | Cardiovascular symptoms | Symptoms | Cardiovascular | 4 |  |
|  | Oedema | Symptoms | Oedema | 4 |  |
|  | Pruritus/Itching | Symptoms | Wound and skin conditions | 4 |  |
|  | Ulcers, pressure sores and wound problems | Symptoms | Wound and skin conditions | 4 |  |
| **Spilsbury, Rosenwax (2017)** | Shortness of breath | Symptoms | Dyspnoea | 11 | Hospital ED rates were 50% lower overall in the intervention group (95% CI: 0.44–0.53). |
|  | Abdominal pain | Symptoms | Pain | 10 |  |
|  | Chest pain | Symptoms | Cardiovascular | 7 |  |
|  | Nausea | Symptoms | Gastrointestinal | 4 |  |
|  | Back pain | Symptoms | Pain | 3 |  |
| **Mercadante, Masedu (2016)** | Dyspnoea | Symptoms | Dyspnoea | 23 |  |
|  | Neurological | Symptoms | Neurological/CNS | 18 |  |
|  | Family fatigue | Patient or carer distress/fatigue |  | 17 |  |
|  | Gastrointestinal Symptoms | Symptoms | Gastrointestinal | 12 |  |
|  | Other | Other/Unknown |  | 11 |  |
|  | Fever | Symptoms | Fever | 7 |  |
|  | Renal failure | Symptoms | Urological/Renal | 5 |  |
|  | Hemorrhage | Acute Event | Blood loss | 4 |  |
|  | Pain | Symptoms | Pain | 2 |  |
|  | Delirium | Symptoms | Cognitive impairment | 2 |  |
| **Batchelor (2015)** | Unknown | Other/Unknown |  | 23 |  |
|  | Fall | Acute Event | Fall | 16 |  |
|  | Fracture | Acute Event | Other medical event | 7 |  |
|  | Foley | Symptoms | Urological/Renal | 5 |  |
|  | Chest pain | Symptoms | Cardiovascular | 5 |  |
|  | Pneumonia | Acute Event | Infection | 4 |  |
|  | Dyspnoea | Symptoms | Dyspnoea | 3 |  |
|  | Mental status change | Symptoms | Cognitive impairment | 2 |  |

^a^ Percentages may exceed 100% because some records allowed for multiple reasons for a single admission, leading to overlapping categories in the data.

^b^ Percentages may be under 100% if only select reasons were reported, either due to the study presenting only the top reasons or because reason collection was incidental to the study.

^c^ The percentages are approximate, as the results were displayed in a bar chart without explicit numerical values.

^d^ COPD exacerbation is categorised under dyspnoea, as it is a hallmark symptom and is cited in the primary article as a key reason for hospital presentation.

1. Martins MdLdC, Pinto S. Analysis of a telephone hotline for palliative care patients at home and their families. International Journal of Palliative Nursing. 2023;29(12):571-7. <https://doi.org/10.12968/ijpn.2023.29.12.571>.

2. DeAngelis J, Lowry MF. Hospital readmissions in hospice patients: evaluation of medication-related causes for readmission. American Journal of Hospice and Palliative Medicine. 2021;38(7):745-9. <https://doi.org/10.1177/1049909120959641>.

3. Gamblin V, Prod'homme C, Lecoeuvre A, Bimbai A, Luu J, Hazard PA, et al. Home hospitalization for palliative cancer care: factors associated with unplanned hospital admissions and death in hospital. BMC Palliative Care. 2021;20(1):24. <https://doi.org/10.1186/s12904-021-00720-7>.

4. Hsu H-S, Wu T-H, Lin C-Y, Lin C-C, Chen T-P, Lin W-Y. Enhanced home palliative care could reduce emergency department visits due to non-organic dyspnea among cancer patients: a retrospective cohort study. BMC Palliative Care. 2021;20(1):1-7. <https://doi.org/10.1186/s12904-021-00713-6>.

5. Cao T, Johnson A, Coogle J, Zuzelski A, Fitzgerald S, Kapadia V, et al. Incidence and Characteristics Associated with Hospital Readmission after Discharge to Home Hospice. Journal of Palliative Medicine. 2020;23(2):233-9. <https://doi.org/10.1089/jpm.2019.0246>.

6. Scheerens C, Pype P, Van Cauwenberg J, Vanbutsele G, Eecloo K, Derom E, et al. Early Integrated Palliative Home Care and Standard Care for End-Stage COPD (EPIC): A Phase II Pilot RCT Testing Feasibility, Acceptability, and Effectiveness. Journal of Pain and Symptom Management. 2020;59(2):206-24.e7. <https://doi.org/10.1016/j.jpainsymman.2019.09.012>.

7. Jessop M, Fischer A, McNeilly A, May A, Good P. Characteristics of community palliative care patients requiring acute admission to hospital. Progress in Palliative Care. 2018;26(2):73-80. <https://doi.org/10.1080/09699260.2018.1453270>.

8. Kao Y-H, Liu Y-T, Koo M, Chiang J-K. Factors associated with emergency services use in Taiwanese advanced cancer patients receiving palliative home care services during out-of-hours periods: a retrospective medical record study. BMC Palliative Care. 2018;17:1-. <https://doi.org/10.1186/s12904-018-0302-8>.

9. Skov Benthien K, Nordly M, von Heymann-Horan A, Rosengaard Holmenlund K, Timm H, Kurita GP, et al. Causes of Hospital Admissions in Domus: A Randomized Controlled Trial of Specialized Palliative Cancer Care at Home. Journal of Pain and Symptom Management. 2018;55(3):728-36. <https://doi.org/10.1016/j.jpainsymman.2017.10.007>.

10. Kaiser F, Rudloff L, Vehling-Kaiser U, Hollburg W, Nauck F, Alt-Epping B. Palliative home care for patients with advanced haematological malignancies—a multicenter survey. Annals of Hematology. 2017;96(9):1557-62. <https://doi.org/10.1007/s00277-017-3045-3>.

11. Spilsbury K, Rosenwax L, Arendts G, Semmens JB. The Association of Community-Based Palliative Care With Reduced Emergency Department Visits in the Last Year of Life Varies by Patient Factors. Annals of Emergency Medicine. 2017;69(4):416-25. <https://doi.org/10.1016/j.annemergmed.2016.11.036>.

12. Mercadante S, Masedu F, Valenti M, Mercadante A, Aielli F. The characteristics of advanced cancer patients followed at home, but admitted to the hospital for the last days of life. Internal and Emergency Medicine. 2016;11(5):713-8. <https://doi.org/10.1007/s11739-016-1402-1>.

13. Batchelor NH. A Quality Improvement Assessment of Emergency Department Visits by Hospice Home Care Patients. Journal of Hospice & Palliative Nursing. 2015;17(5):442-9. <https://doi.org/10.1097/NJH.0000000000000182>.
